# Comparison of the Results of Modeling Pulmonary Fibrosis in Sprague Dawley Rats by Intratracheal Administration of Bleomycin in the Form of Sulfate and Chloride at a Dose of 3 mg/kg

**DOI:** 10.3390/ph17101360

**Published:** 2024-10-11

**Authors:** Elena A. Tukhovskaya, Yulia A. Palikova, Mariya S. Severyukhina, Alina M. Ismailova, Victor A. Palikov, Gulsara A. Slashcheva, Natalya A. Borozdina, Evgeniy S. Mikhaylov, Irina N. Kravchenko, Vitaly A. Kazakov, Ekaterina N. Kazakova, Elena A. Kalabina, Ekaterina A. Rasskazova, Maxim V. Shinelev, Dmitry I. Rzhevsky, Vladimir A. Rykov, Igor A. Dyachenko, Arkady N. Murashev

**Affiliations:** Shemyakin-Ovchinnicov Institute of Bioorganic Chemistry (Branch), Russian Academy of Sciences, Prospekt Nauki, 6, Pushchino 142290, Russia

**Keywords:** pulmonary fibrosis, bleomycin, animal model of pulmonary fibrosis

## Abstract

**Background/Objectives**: Intratracheal administration of bleomycin (BLM) to laboratory rodents is a standard, widely used technique used to model pulmonary fibrosis (PF). BLM, as a modeling agent, is produced mainly in the form of two salts—sulfate and chloride. We compared the results of modeling PF in SD rats by intratracheal administration of BLM sulfate and BLM chloride. **Methods**: Healthy mature male SD rats were used. PF was modeled by intratracheal administration of BLM sulfate and BLM chloride at a dose of 3 mg/kg. The criteria for the development of PF included body weight gain, changes in respiratory parameters, relative lung weight, cellular composition of broncho-alveolar fluid (BALF), histological assessment of the severity of PF with trichrome Masson staining. **Results**: Intratracheal administration of both BLM salts led to the development of pronounced PF, which was determined by changes in all of the measured parameters relative to control animals. There were no significant differences between the BLM sulfate and BLM chloride groups in body weight gain, hydroxyproline content, and histological evaluation. However, significant differences were identified in the cellular composition of BALF—a significant increase in alveolar macrophages and neutrophils levels in animals treated with BLM sulfate. **Conclusions**: Intratracheal administration of both BLM salts led to the development of severe PF; however, the inflammatory process in animals receiving BLM sulfate was more pronounced and prolonged than in animals receiving BLM chloride, which in the former, when observed more than 21 days after modeling, can lead to more severe PF.

## 1. Introduction

PF is usually observed in the advanced stages of various pulmonary diseases, such as interstitial lung disease and acute respiratory distress syndrome [1,2]. PF is characterized by excessive accumulation of fibrous tissue replacing healthy lung tissue, leading to progressive impairment of pulmonary function and death, on average, 1–2 years after diagnosis [3,4]. The search and study of new drugs for the treatment of PF is an urgent task of medical science. For the preclinical studies of such drugs, a model of PF caused by intratracheal administration of BLM to laboratory rodents is widely used. BLM is either a mixture of glycopeptide antibiotics (A2, B2, and other fractions) or isolated fractions isolated from the Streptomyces verticillus strain. BLM, in combination with other cytostatics and/or radiation therapy, is used to treat a number of malignant tumors. The primary mechanism of action is associated with the ability to cause fragmentation of DNA molecules in the form of single-strand or double-strand breaks, which stops cell division. A side effect of BLM is the development of pulmonary toxicity, namely the development of PF, characterized by inflammatory infiltration into pulmonary endothelial cells with a subsequent increase in their collagen content [5]. The presence of this side effect of BLM has led to its widespread use for modeling PF in laboratory animals. Using this model of PF, the mechanisms of development of PF, which are not yet fully understood, are studied, as well as the effectiveness of new drugs for the treatment of PF. BLM is available in the form of hydrochloride or sulfate; a unit of bleomycin is equal to the formerly used milligram activity. To model PF, mice and rats are used that are intratracheally injected with a BLM solution. The BLM doses most often used to model PF are 3 mg/kg and 5 mg/kg [6,7,8,9,10]. Let us consider the general scheme of an experiment with modeling PF in laboratory rodents. Animals are divided into groups containing a minimum of five animals. Typically, the groups used in the study are control-sham (saline administration in the modeling), control-disease (using only the injurious agent for modeling followed by administration of the vehicle), test drug (injurious agent for modeling with treatment with the test drug), comparator drug (injurious agent for modeling with treatment with a comparator drug). Either pirfenidone [10,11] or dexamethasone [12,13] is used as a comparator drug (Table 1).

Thus, in the simplest study of the effectiveness of a drug for the treatment of PF, a minimum of 15 animals are needed, in which PF will be modeled by administering BLM. For rats with an average body weight of 300 g using a BLM dose of 3 mg/kg, taking into account a 25% reserve, a minimum of 17 mg of BLM will be required. When using a dose of 5 mg/kg, 28 mg of BLM will be required, respectively. And when studying the test drug in several doses, these quantities will increase in proportion to the number of animals (minimum five in each group). For mice weighing an average of 30 g, the amount of BLM required to model PF in rats should be divided by 10. BLM is used both in the form of a chemical reagent and in the form of a drug (Table 2). It is much less economically feasible to use BLM in the form of a chemical reagent for modeling PF than in the form of a medicinal product. Thus, the cost of the chemical reagent BLM sulfate produced by Cayman Chemicals (one of the cheapest) is 40 euros per 5 mg [14], and the cost of the BLM chemical reagent in the form of hydrochloride produced by LKT Laboratories is 493 euros for 5 mg [14]. Medicines intended for human therapy will cost much less than chemical reagents. Thus, in the USA, the cost of 15 units of BLM ranges from 41 to 68 USD [15]. Thus, when using BLM in the form of a chemical reagent, the economic burden on researchers will be more significant compared to using BLM in the form of a pharmaceutical preparation. As mentioned earlier, to model pulmonary fibrosis, BLM is used in the form of two salts—hydrochloride and sulfate. Table 2 presents a selection of the use of BLM in the form of a reagent or in the form of a pharmaceutical preparation, indicating the type of animal.

As can be seen from the table, in both mice and rats, more than half of the cases of use of BLM in the form of one of the salts are pharmaceutical preparations. As for the frequency of use of one of the salts, both salts are used with approximately equal frequency in both rats and mice. Thus, researchers prefer to use BLM for modeling PF in the form of a pharmaceutical preparation—a ready-to-dissolve lyophilisate with a high degree of purification and a lower cost relative to a chemical reagent.

The purpose of our study was to examine whether there are significant differences in the results of modeling PF in male SD rats using two BLM salts—BLM chloride and BLM sulfate in the form of pharmaceuticals.

## 2. Results

### 2.1. Body Weight Gain

**Figure 1:**
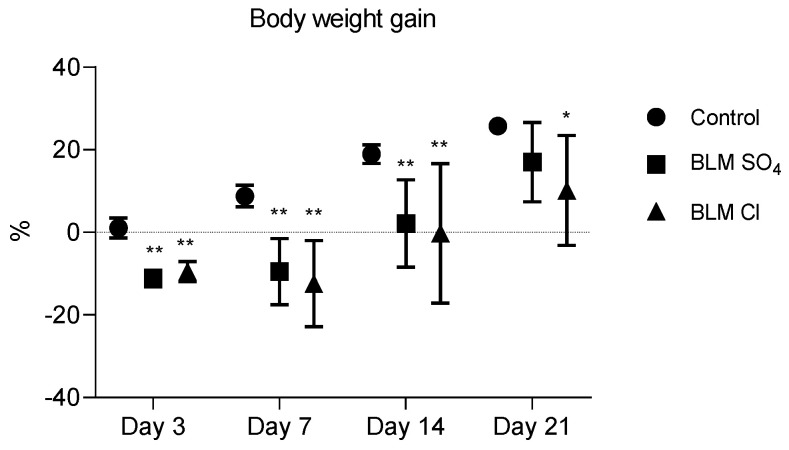
Body weight gain relative to day 1 of the study (before PF modeling). * *p* ≤ 0.05, ** *p* ≤ 0.01 relative to the control group ** *p* ≤ 0.01 relative to the control group in accordance with Mann–Whitney U-test in pairwise comparison. Data are presented as means ± standard deviation.

Body weight gain was significantly reduced relative to the control group in both groups in which pulmonary fibrosis was modeled until the 14th day. On day 21, a significant decrease in body weight gain was maintained only in the group that received BLM chloride to model pulmonary fibrosis (Figure 1).

### 2.2. External Respiration Parameters (Spirometry)

The respiratory rate in both experimental groups receiving two BLM salts relative to the 1st day of the study increased significantly in both experimental groups by the 7th day (by 89 ± 50% in the group receiving BLM sulfate and by 184 ± 35% in the group receiving BLM chloride), and this increase persisted until the 21st day of the study (by 59 ± 34% in the group receiving BLM sulfate and by 81 ± 27% in the group receiving BLM chloride).

**Figure 2:**
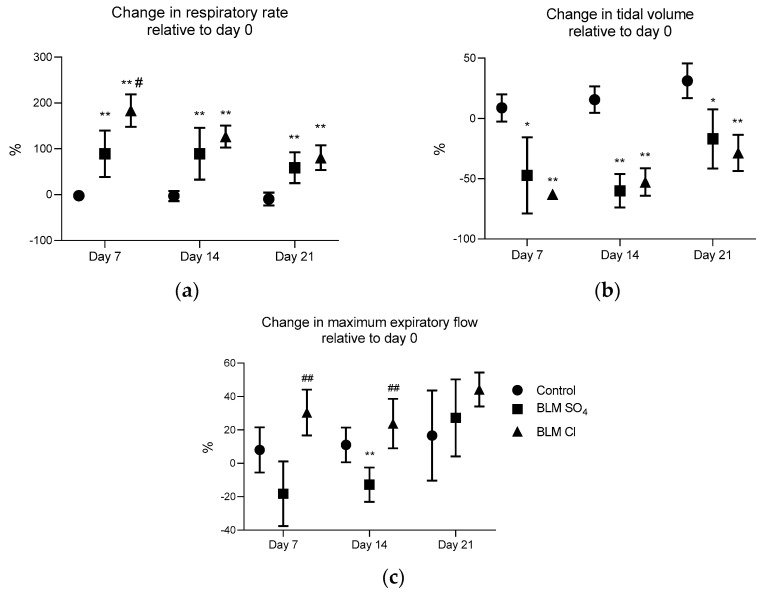
Changes in spirometry parameters relative to day 1 (before PF modeling) on days 7, 14, and 21. (**a**) Change in respiratory rate, %; (**b**) change in tidal volume, %; (**c**) change in maximum expiratory flow, %. * *p* ≤ 0.05, ** *p* ≤ 0.01 relative to the control group, # *p* ≤ 0.05, ## *p* ≤ 0.01 relative to the BLM sulfate group in accordance with Mann–Whitney U-test in pairwise comparison. Data are presented as means ± standard deviation.

The relative respiratory rate was significantly different in both experimental groups relative to the control group on all measurement days. In addition, this indicator in the group receiving BLM chloride was significantly higher than that for the group receiving BLM sulfate on day 7 of the study (89 ± 50% in the group receiving BLM sulfate and 184 ± 35% in the group receiving BLM chloride) (Figure 2a)

The relative tidal volume significantly decreased in the groups receiving both BLM sulfate and BLM chloride compared to the control group, starting from day 7 and remained reduced until the end of the intravital phase until day 21 after modeling pulmonary fibrosis (Figure 2b).

The relative maximum expiratory flow on day 7 differed diametrically between the experimental groups. Thus, in the group receiving BLM sulfate, the maximum expiratory flow decreased, and in the group receiving BLM chloride, on the contrary, it increased relative to the control (−18 ± 19% in the BLM sulfate group versus 30 ± 14% in the BLM chloride group). Similar differences between experimental groups were observed on day 14 (−13 ± 10% in the BLM sulfate group versus 24 ± 15% in the BLM chloride group). At day 21, the relative maximal expiratory rate did not differ between groups (Figure 2c).

### 2.3. Relative Lung Weight

**Figure 3:**
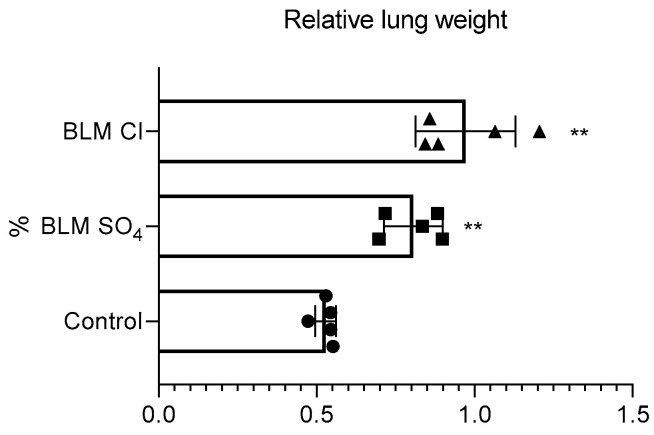
Relative lung weight, % (relative to body weight at necropsy). ** *p* ≤ 0.01 relative to the control group in accordance with Mann–Whitney U-test in pairwise comparison. Data are presented as means ± standard deviation. Individual values are presented as triangles for BLM Cl, squares for BLM SO_4_ and circles for control.

Relative lung weight was significantly increased in both experimental groups receiving both BLM sulfate and BLM chloride compared to the control group (Figure 3).

### 2.4. BALF Cytology

In the BALF, the total concentration of cells counted in the Goryaev chamber was significantly increased in the group receiving BLM sulfate, both relative to the control and relative to the group receiving BLM chloride (2.57 ± 1.17 × 10^5^ in the control group, 14.50 ± 5.29 × 10^5^ in the BLM sulfate group, 4.65 ± 2.28 × 10^5^ in the BLM chloride group). The absolute number of alveolar macrophages was significantly increased in the group receiving BLM sulfate relative to the group receiving BLM chloride, while in both of these groups, the absolute number of macrophages was significantly higher compared to the control group (2.53 ± 1.7 × 10^5^ in the control group, 13.25 ± 5.15 × 10^5^ in the BLM sulfate group, 4.31 ± 2.11 × 10^5^ in the BLM chloride group). At the same time, the relative count of alveolar macrophages in both groups that received BLM both in the form of sulfate and in the form of chloride was reduced in comparison with the control group (98.1 ± 1.1% in the control group, 90.6 ± 5.6% in the BLM sulfate group, 92.6 ± 1.4% in the BLM chloride group) due to an increase in the proportion of neutrophils and lymphocytes (Table 2). The relative count of segmented neutrophils compared to the control group increased in both groups receiving BLM sulfate and chloride and was also significantly higher in the group receiving BLM sulfate compared to the group receiving BLM chloride (0.2 ± 0.2% in the control group, 4.1 ± 1.8% in the BLM sulfate group, 2.7 ± 0.9% in the BLM chloride group). The absolute number of lymphocytes, in comparison with the control group, was significantly higher in both groups receiving BLM, while in the group receiving BLM sulfate, this value was significantly higher in comparison with the group receiving BLM chloride (0.037 ± 0.025 × 10^5^ in control group, 0.663 ± 0.175 × 10^5^ in the BLM sulfate group, 0.220 ± 0.138 × 10^5^ in the BLM chloride group), while the relative count of lymphocyte between BLM sulfate and BLM chloride did not differ but was higher for both groups compared to the control (1.7 ± 1.2% in the control group, 4.9 ± 1.6% in the BLM sulfate group, 4.6 ± 1.2% in the BLM chloride group) (Table 3).

### 2.5. Hydroxyproline ELISA

**Figure 4:**
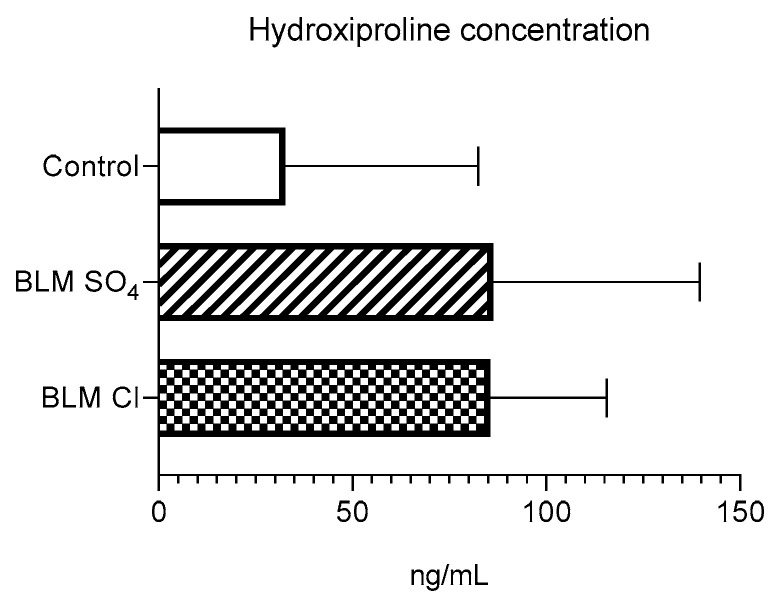
Hydroxyproline concentration, ng/mL. Data are presented as means + standard deviation.

Left lung hydroxyproline concentrations measured by ELISA were not statistically different between groups but were visually higher in both groups receiving BLM sulfate and BLM chloride (Figure 4).

### 2.6. Histological Evaluation

**Figure 5:**
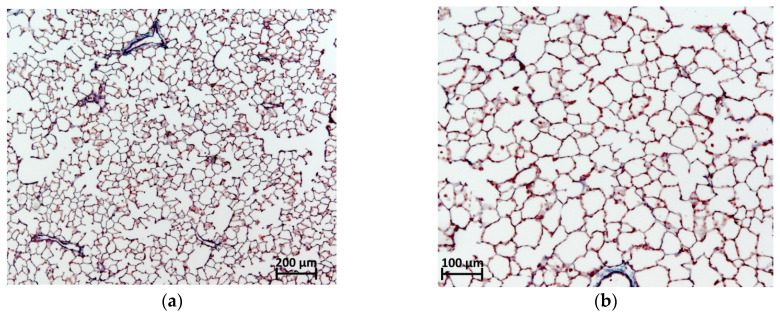

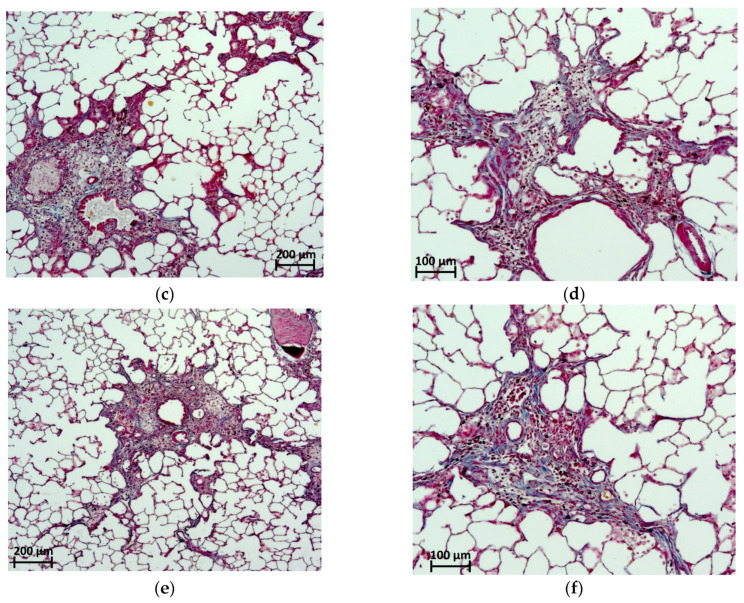
Microphotographs of lung fragments 21 days after modeling of PF through a single intratracheal injection of BLM at a dose of 3 mg/kg: (**a**,**b**)—lung fragments after a single intratracheal injection of saline in a volume of 0.5 mL/kg; (**c**,**d**)—fragments of the lung with focal fibrotic changes after a single intratracheal injection of BLM sulfate at a dose of 3 mg/kg; (**e**,**f**)—fragments of the lung with focal fibrotic changes after a single intratracheal injection of BLM chloride at a dose of 3 mg/kg. Masson trichrome staining. Magnification 50× (**a**,**c**,**e**) and 100× (**b**,**d**,**f**).

In the examined right lung of animals that received intratracheal BLM both in the form of sulfate and in the form of chloride at a dose of 3 mg/kg, focal phenomena of PF were noted. Foci of fibrous tissue was most often localized in the hilar peribronchial spaces, less often in the pulmonary acini without an obvious connection with the bronchi, and in isolated cases, subpleurally (Figure 5). By the 21st day of observation, numerous fibroblasts and mononuclear cells were visualized in the PF lesions; collagen fibers were characterized by a sufficient degree of maturity.

**Figure 6:**
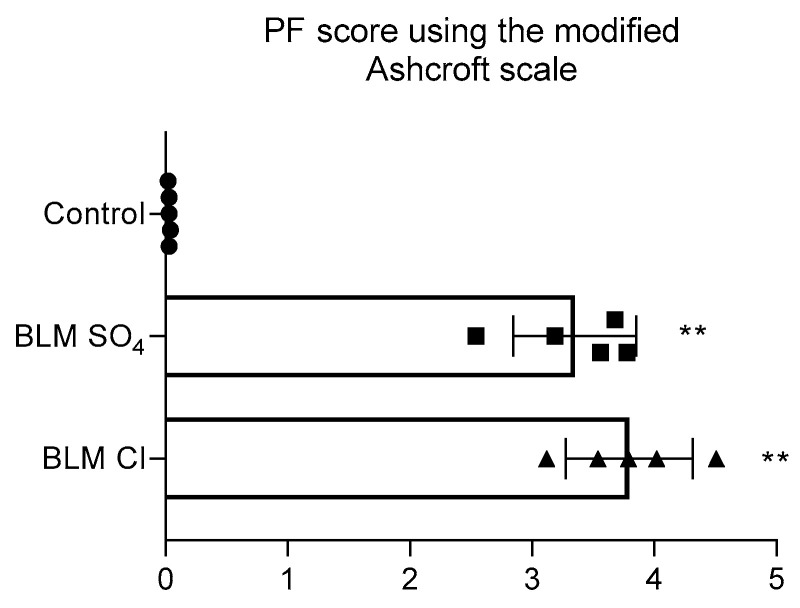
Assessment of the severity of pulmonary fibrosis using the modified Ashcroft scale. ** *p* ≤ 0.01 relative to the control group in accordance with Mann–Whitney U-test in pairwise comparison. Data are presented as means + standard deviation. Individual values are presented as triangles for BLM Cl, squares for BLM SO_4_ and circles for control.

Preserved pulmonary acini in the lungs with focal fibrous changes were distinguished by an emphysematous dilated lumen with thinned interalveolar septa. The average PF score according to the semi-quantitative 8-point modified Ashcroft scale in the group of animals receiving BLM sulfate corresponded to 3.35 ± 0.50 points, and those receiving BLM chloride corresponded to 3.80 ± 0.52 points; no differences in the severity of fibrosis were observed between these two groups, while from the control, the differences in both groups receiving BLM were statistically significant. On the 21st day, after a single intratracheal injection of saline in a volume of 0.5 mL/kg, no deviations from the norm were observed in the lungs of rats (Figure 6).

## 3. Discussion

Our study shows that intratracheal administration of BLM in the form of pharmaceutical preparations at a dose of 3 mg/kg, both in the form of sulfate and chloride, leads to the formation of pronounced PF in animals by the 21st day.

In our study, we used the 21st day as the endpoint to evaluate the result of the simulation, namely, the fully formed PF. When studying the dynamics of PF development, one can see the stages of fibrosis formation and understand that by the 21st day, pulmonary tissue fibrosis is fully formed [45,48]. This is confirmed in our study by the clinical picture characteristic of PF.

Young animals used in preclinical studies, aged 8–12 weeks, are characterized by a steady increase in body weight. In our study, in animals in which pulmonary fibrosis was modeled by administration of BLM sulfate or BLM chloride, the rate of body weight gain steadily decreased from the 3rd day to the 21st day. Adults with pulmonary diseases such as interstitial pneumonia and chronic obstructive pulmonary disease (COPD), which ultimately lead to the development of pulmonary fibrosis, are characterized by a pronounced decrease in body weight—pulmonary cachexia [61]. Pulmonary cachexia is a significant risk factor for mortality in people with COPD [62]. In most studies modeling PF in animals, body weight gain is reduced relative to control animals [47,48,49,55]. Body weight in pulmonary cachexia is reduced due to muscle atrophy, which, in turn, is a consequence of the inflammatory process in the lungs, which entails an acute phase response, including adaptive metabolic mechanisms that increase protein degradation in the muscles [61,63]. It has been shown that when pulmonary function is impaired, energy metabolism changes—ATP is produced anaerobically instead of aerobically [64]. The anaerobic method of producing ATP is less efficient than the aerobic method, which shifts cellular energy metabolism, reducing the level of aerobic enzymes. A quarter of patients with COPD have increased resting metabolism, which is a response to an inflammatory state and is a sign of pulmonary cachexia [65]. Thus, by showing a persistent decrease in body weight gain in animals receiving both BLM salts in our study, we confirmed the presence of one of the most important signs of severe lung damage—pulmonary cachexia.

When assessing external respiratory function in patients with PF in clinical practice using spirometry, an increase in respiratory rate and a decrease in tidal volume are observed [66,67,68], which was also demonstrated in our study for both BLM salts. An increase in respiratory rate has also been shown in other animal studies [48]. The indicator of maximum expiratory volume, similar to the forced vital capacity of the lungs, differed between the two salts—on the 7th and 14th days, the values of this indicator differed diametrically—BLM sulfate caused a decrease, and BLM chloride, on the contrary, caused an increase in the change in this indicator relative to the initial values. Fujita et al., 2022, showed that a decrease in the peak expiratory flow rate is a prognostic factor for an unfavorable outcome of the disease [69], so we can talk about a more detrimental effect of BLM sulfate on the vital capacity of the lungs up to the 14th day after modeling; however, by the 21st day, this indicator increased and did not differ from the control for either BLM sulfate or BLM chloride.

We showed that modeling of PF using both BLM salts led to an increase in the relative lung weight of animals by day 21. This increase occurs due to the development of interstitial inflammation in the early stages of PF formation, during which neurophils migrate into the interstitial space and the level of proinflammatory mediators increases, there is a subsequent thickening of the alveolar walls and the interstitium, and fibrosis progresses [7,70,71,72,73,74]. The fibrotic lung increases in mass, and the relative lung mass further increases due to the development of pulmonary cachexia (decreased muscle mass), which is also observed in other studies modeling PF [47].

In the work of Kadam et al., 2024 [49], when modeling PF by intratracheal administration of BLM sulfate at a dose of 2 mg/kg, the maximum content of neutrophils in the broncho-alveolar fluid occurred on the 3rd day after modeling, significantly decreasing by the 14th day. In the same study, the authors showed that by the 14th day after the simulation, there was an increase in the absolute number of alveolar macrophages. The same data have been shown by other researchers [35]. In our study, on the 21st day after modeling, macrophages in absolute and percentage terms constituted the main pool of cells in BALF in all groups, including the control group, which is consistent with the composition of BALF in humans [75]. In both groups with modeling of fibrosis with BLM sulfate and BLM chloride, a significant increase in the absolute number of macrophages was observed in comparison with the control. Moreover, in absolute terms, the total concentration of cells, and, accordingly, the number of macrophages, was three times higher in animals that were administered BLM sulfate compared to animals that received BLM chloride. Alveolar macrophages play a critical role in tissue healing [76]. In the acute phase of inflammation (up to the 8th day after BLM administration), proinflammatory M1 macrophages predominate in the lungs, producing proinflammatory cytokines [77]. However, under conditions of chronic inflammation, they transform into the anti-inflammatory form of M2 macrophages [78,79]. This form of macrophages secretes growth factors such as GF-β, fibroblast growth factor (FGF), platelet-derived growth factor-α (PDGFα), and insulin-like growth factor 1 (IGF1), and vascular endothelial growth factor (VEGF) [80] promotes wound healing and has profibrogenic properties. The absolute number of neutrophils in animals receiving BLM sulfate was five times higher than that for animals receiving BLM chloride. At the same time, there was also a one-and-a-half-fold superiority of BLM sulfate in the relative number of neutrophils, and in comparison with the control, both the absolute and relative number of neutrophils in both groups receiving BLM in the form of sulfate and chloride was increased. An increased level of neutrophils and lymphocytes on the 21st day after administration indicates an ongoing inflammatory process. In animals with PF modeled by the introduction of BLM, the development of fibrosis occurs faster than in humans under the influence of pathological factors, so inflammation plays a large role in animal models. With the development of acute inflammation after BLM administration, under the influence of pro-inflammatory chemokines such as IL8, neutrophil infiltration of damaged tissues occurs. Neutrophils release pro-inflammatory cytokines and free forms of oxygen [76,81]. It has been shown in mice that blocking neutrophil chemotaxis by inhibiting IL-8 reduces the development of BLM-induced PF [82]. Accordingly, in our study, BLM sulfate causes more severe and prolonged inflammation, which may give a more aggravated picture of PF when observed longer than 21 days. Thus, in the work of Bonatti et al., 2023, where PF was modeled in rats by administration of BLM sulfate 2 mg/kg, Sigma, USA, with double administration on day 0 and day 4, a gradient increase in the level of hydroxyproline can be observed from days 14 to 56. The histological picture of the lungs, judging by the figures, also worsens—so on the 21st day, the lungs are represented by fibrotic foci of low density and low emphysematous dilation of the pulmonary acini, and on the 56th day, fibrosis is represented by more dense foci, and the pulmonary acini are pathologically emphysematous dilated. That is, fibrosis worsens compared to the 21st day of observation [22]. In the work of Chu et al., 2019, unilateral pulmonary fibrosis of the left lung was modeled in rats by administering BLM hydrochloride from Nippon Kayaku, Japan, at a dose of 8 mg/kg, followed by observation for 49 days. Histological assessment of the lung over time showed no worsening of the morphological picture of PF from the 21st to the 49th day after the simulation. Additionally, there was no increase in collagen content in the lungs from days 21 to 49 [48]. In people with idiopathic PF, an inverse relationship has been shown between a decrease in forced vital capacity and an increased content of neutrophils in the BALF [83,84,85].

A similar feedback was observed in our study—the maximum expiratory flow in animals receiving BLM sulfate was significantly reduced on the 7th and 14th days of the study, with a significant increase in the content of neutrophils in the BALF on the 21st day. Thus, we can talk about a likely more severe effect of BLM sulfate, which manifests itself after the 21st day of observation.

Determination of hydroxyproline content in lung tissues affected by PF is a generally accepted method for assessing the degree of lung damage when modeling BLM-induced PF in animals. Hydroxyproline is a degradation product of type 1 collagen, which forms the basis of fibrous replacement tissue; therefore, assessment of its level in lung tissue is an index indicating the degree of PF [40,86]. Our work shows that BLM chloride and BLM sulfate, administered intratracheally at a dose of 3 mg/kg, lead to an increase in the level of hydroxyproline compared to control animals, which indicates the formation of fibrosis in the lung tissues. Histological evaluation confirms the formation of fibrosis in the lung tissues for both BLM salts. However, as mentioned above, the content of hydroxyproline or collagen after the 21st day of observation may differ between animals in which pulmonary fibrosis was modeled with BLM sulfate (continues to increase) and BLM (remains unchanged) [22,48].

For histological assessment, most researchers use Masson trichrome staining and subsequent analysis of histological preparations using a scoring system—the Ashcroft scale. This scale, in its original version [87], is a system where the gradation of points from the mildest degree of severity to the most severe is placed on a 6-point scale and is used by a number of researchers [30,45]. However, many researchers have modified this scale to increase the accuracy of assessing the severity of pulmonary fibrosis. We used a modified scale that has an eight-point gradation [7,26,34,88]. When semiquantitatively assessing the severity of PF in the lungs using the Ashcroft scale, our study showed an increase in the score that was equal for both BLM salts (0.03 ± 0.01 for control, 3.35 ± 0.50 for BLM sulfate, 3.80 ± 0.52 for BLM chloride), which is typical for studies animal models of PF [45,47].

When assessing the histological picture, in most studies, hilar peribronchial fibrosis is observed, regardless of the method of administration of the damaging BLM agent (intravenous, through the nose, and intratracheal) [26,35]. We chose the most commonly used method of administration, namely intratracheal, which we modified by hyperventilation of the lungs for better distribution of the BLM sulfate or BLM chloride solution over the inner surface of the lungs [89,90]. In addition to improving the uniformity of distribution of the damaging agent, hyperventilation is intended to serve as an additional damaging factor that aggravates inflammation and the subsequent development of pulmonary fibrosis [91,92]. However, in spite of everything, the picture of PF we observed did not represent peripheral damage—fibrosis was localized, as in other studies, in the hilar and peribronchial spaces.

Since the histological pattern we observed was assessed on day 21 after PF simulation (the endpoint most common for assessing the severity of BLM-induced pulmonary fibrosis), we did not observe differences between BLM chloride and BLM sulfate in either the morphological assessment or the semiquantitative assessment according to the modified Ashcroft scale. However, in some studies in which BLM chloride or BLM sulfate were used, where observation was carried out over time longer than 21 days, one can see a worsening of the histological picture in animals in which pulmonary fibrosis was modeled with BLM sulfate, and a stable lesion was seen in animals in which pulmonary fibrosis was modeled with BLM chloride.

## 4. Materials and Methods

### 4.1. Animals

The study used 15 mature male SD rats, 8–10 weeks old. Animals were obtained from the Pushchino nursery of laboratory animals (Pushchino, Russia). All procedures and manipulations with animals were approved by the Committee for Control over Care and Use of Laboratory Animals of BIBCh RAS (IACUC) (protocol number 928/23 from 03/14/2023) and were carried out in accordance with the EU Directive 2010/63/EU. After receiving from the nursery, the animals were adapted within 7 days. During this period, the animals were monitored for signs of deviation in health status. Animals without signs of health deviations were selected for the experiment (clinical examination was performed). Animals were randomly divided into groups using organ weight as a criterion so that the average weight of animals did not differ between groups. Each animal was assigned an individual number, according to which the animal was marked with a puncture of the auricle. During the study, the animals were kept under controlled environmental conditions in a barrier zone with a “clean” and “dirty” corridor system with controlled environmental conditions (temperature 20–24 °C, relative humidity 30–55%, 12 h light cycle; 08:00–20:00—“day”, 20:00–08:00—“night”; 10-fold change in air volume in the room per hour). The animals received enough food ad libitum for laboratory mice and rats according to the Velaz FORTI 1324 Maintenance Diet (Altromin Spezialfutter GmbH & Co KG, Im Seelenkamp 20, D-32791 Lage, Germany).

### 4.2. Design Description

The animals were divided into groups of 5 animals per group: group No. 1—animals that received intratracheal saline solution, group No. 2—animals that received intratracheal BLM sulfate at a dose of 3 mg/kg, group No. 3—animals that received intratracheal BLM chloride at a dose of 3 mg/kg. During the lifetime phase of the study, clinical signs of abnormal health status, body weight, and respiratory parameters were recorded in animals. Animals were euthanized on the 22nd day of the study. At necropsy, the lungs of all animals were isolated and weighed, and broncho-alveolar lavage (BAL) was performed on the left lung to assess the number of cells and cellular composition of the broncho-alveolar lavage fluid (BALF) [18,24]. After BAL, the left lung was separated from the bronchus and divided into two parts; each part was placed in a microcentrifuge tube and frozen at −70 °C for further analysis of hydroxyproline levels in the tissue. The right lung was fixed in 10% neutral formalin after filling it with fixative for further histopathological examination. In sections of the right lung, the severity of fibrosis was assessed using specific Masson trichrome staining.

### 4.3. Modeling of PF

PF was modeled as described previously [9] by a single intratracheal administration of a BLM solution at a dose of 3 mg/kg in a volume of 0.5 mL/kg, followed by hyperventilation. The animals were anesthetized with an intramuscular injection of the Telazol^®^/Xyla^®^ mixture at a dose of 40 mg/kg + 10 mg/kg and were fixed on the surgical table in a supine position by the hind legs and upper incisors. The lower jaw was pulled back, and the oral cavity was opened. A metal probe with a ball at the end (a probe for intragastric administration to mice) was inserted along the surface of the tongue into the oral cavity. Having overcome the resistance of the vocal cords (while exhaling), the probe was inserted into the trachea. A polyethylene catheter (PE-10, outer diameter 0.60 mm) was inserted into the probe hole and inserted to a pre-measured depth (from the incisors to the middle of the sternum). A syringe with a BLM solution (or saline in the control group) was connected to the catheter, and holding the animal in an upright position, a slow injection was made in a volume of 0.5 mL/kg. Hyperventilation of the lungs was performed in all animals immediately after intratracheal administration. An Ugo Basile 7025 device for artificial ventilation of the lungs was connected to the probe located in the trachea. The set parameters for pulmonary hyperventilation were tidal volume of 30 mL/kg, respiratory rate of 60 times/min, exposure time of 10 min. After completion of the procedure, the animals were returned to the housing cage under constant observation until recovery from the anesthesia state.

### 4.4. Body Weight Gain

Body weight was recorded when the groups were formed on day 1 (before modeling), day 3, day 7, day 14, and day 21. After the end of the intravital phase of the study, body weight gain was calculated relative to body weight on day 1 of the study.

### 4.5. External Respiration Parameters (Spirometry)

Respiratory parameters (respiratory rate, tidal volume, and maximal expiratory flow) were measured on study days 1 (before modeling), 7, 14, and 21 using the FE141 Spirometer rodent spirometry unit of the PowerLab 8/35 computerized system (ADInstruments Pty Ltd., Bella Vista, Australia). Within the groups, changes in breathing parameters were calculated relative to the measurement results on day 0 of the study before modeling, and the differences in these relative indicators between the groups were assessed.

### 4.6. Euthanasia and Necropsy

Animals were anesthetized with an intramuscular injection of Telazol^®^/Xyla^®^ mixture at a dose of 40 mg/kg + 10 mg/kg, after which laparotomy was performed, and blood was collected from the caudal vena cava using a 5 mL syringe and a G25 needle. The animals died in a state of anesthesia from exsanguination. Necropsy of all animals was performed, which included examination of the external surface of the body, all orifices, the chest, abdominal and pelvic cavities, including internal organs. Lungs with trachea were removed for weighing, BAL, tissue sampling, and fixation for histopathological examination.

### 4.7. Relative Lung Weight

After isolation and purification of the lungs, they were weighed together with the trachea. The relative weight of the lungs relative to the body weight of the animal before euthanasia was determined.

### 4.8. BAL Performing

After weighing the lung–trachea complex, the right bronchus was ligated, and BAL was performed to obtain BALF. BAL was performed by injecting phosphate-buffered saline (PBS) solution in a volume of 2 mL into the lung through the trachea using a 2 mL syringe with a blunt needle and draining the resulting wash by gravity into a 15 mL tube. The procedure was performed three times, with a total volume of PBS for BAL fluid of 6 mL. The LVFL was divided into two parts, one of which was transferred for counting the total number of cells in the washout in the Goryaev chamber. The second was transferred for centrifugation and subsequent preparation of a smear to determine the cellular composition per 100 cells. In the Goryaev chamber, the number of nucleated cells was counted after erythrocyte lysis, performed using a tenfold dilution of the resulting wash with 4% acetic acid. The concentration of nucleated cells (including dilution) was used to recalculate the percentage of leukocyte subpopulations counted on the smear. For the smear, half of the wash was centrifuged in an Eppendorf 5804 R centrifuge at 3000 rpm, 4 °C for 15 min. The supernatant was collected, the pellet was suspended with 3 μL of fetal bovine serum, and a smear was made. The smear was stained, according to Pappenheim. The cellular composition of the stained smears was analyzed: the percentage of alveolar macrophages, segmented neutrophils, band neutrophils, eosinophils, and lymphocytes was calculated per 100 cells.

### 4.9. BALF Cytology

Half of the BALF was taken to calculate the concentration of nucleated cells. To accomplish this, a tenfold dilution of BALF was performed in 4% acetic acid to lyse erythrocytes. The liquid sample obtained after lysis was filled into the Goryaev chamber, and the concentration of nucleated cells was calculated, taking into account the dilution. The other half of the BALF was used to prepare a smear and calculate the percentage of leukocyte subpopulations, for which the BALF was centrifuged in an Eppendorf 5804 R centrifuge at 3000 rpm, 4 °C for 15 min. The supernatant was collected, the pellet was suspended with 3 μL of fetal bovine serum, and a smear was made. The smear was stained, according to Pappenheim. The cellular composition of the stained smears was analyzed: the percentage of alveolar macrophages, segmented neutrophils, band neutrophils, eosinophils, and lymphocytes was calculated per 100 cells. The concentration of nucleated cells was used to convert the percentage of leukocyte subpopulations counted on a smear into the absolute concentration of each subpopulation.

### 4.10. Taking Samples of Lung Tissue for Subsequent Analysis of Hydroxyproline Content

After performing BAL on the left lung, the left bronchus was ligated, the left lung was separated from the bronchus, weighed, divided into two parts, and frozen at −70 °C; this was performed for each part separately in pre-weighed microcentrifuge tubes for further analysis of the level of hydroxyproline in the lung tissue

### 4.11. Sample Preparation for Analysis of Hydroxyproline in the Lung

Frozen lung samples were thawed and then homogenized with an MT-30K manual homogenizer; the mass of the homogenate was determined by subtracting the mass of the empty tube from the mass of the test tube with the resulting homogenate. The homogenate was frozen at −70 °C, then thawed again, and a PBS solution was added in a ratio of 1:12 (weight/volume); the resulting mixture was transferred into a 15 mL test tube and mixed, and then the mixture was frozen and thawed twice. After the second thawing of the homogenate + PBS mixture, it was centrifuged in an Eppendorf 5804 R centrifuge at 3000 rpm and 4 °C for 15 min and then immediately re-centrifuged at 5000 rpm and 15 °C for 15 min. The resulting supernatant was concentrated in special concentrator tubes JetSpinTM Centrifugal Filter, 5 mL, 100,000 MWCO, PES, Non-sterile, BIOFIL, China. After washing the tube with PBS, the supernatant from the homogenate + PBS mixture was poured into the tube and centrifuged at 3500 rpm, 21 °C. Centrifugation time for all samples was different (from 30 to 140 min). The samples were centrifuged to twice the volume relative to the mass of the homogenate (for example, the mass of the homogenate is 0.3 g—centrifugation to a volume of 0.6 mL).

### 4.12. Hydroxyproline Enzyme Immunoassay (ELISA)

Analysis of the obtained sample from the lung homogenate for hydroxyproline content was performed using the Rat Hyp (Hydroxyproline) ELISA Kit, ELK Biotechnology using an immunological plate spectrophotometer Multiskan™GO 1.01.12, Termo Scientific, and measured at 450 nm.

### 4.13. Histological Evaluation

Histological evaluation was performed on sections stained with Masson’s trichrome stain (Masson Trichrome stain, Bio-Optica, Italy). Masson staining is used to identify the structural components of connective tissue (collagen and reticular fibers are stained blue). Histological preparations were studied using conventional light microscopy on an AxioScope.A1 microscope (Carl Zeiss, Germany). Microphotographs of histological preparations were obtained using a high-resolution camera, Axiocam 305 color (Carl Zeiss, Germany), using ZEN 2.6 lite software (Carl Zeiss, Germany). Lung sections stained using the Masson method were examined at 20× objective magnification for the severity of fibrotic changes using a modified semi-quantitative 8-point Ashcroft scale [88]. The final score for pneumofibrosis is the average value of the degree of fibrotic changes in the lung in all assessed fields of view and in all sections of the organ.

### 4.14. Statistical Evaluation

For all data, the mean and standard deviation were calculated using the Excel program. To assess differences between groups, pairwise comparison was used using the Mann–Whitney U-test using the Statistica for Windows v.7 program. Significance was established at *p* ≤ 0.05.

## 5. Conclusions

The use of intratracheal administration of a BLM solution at a dose of 3 mg/kg in the form of sulfate or chloride (both in the form of pharmaceuticals) to model PF in rats does not have significant differences in the modeling results on the 21st day after administration, neither in terms of body weight gain nor in histology. However, the cytological composition of the BALF shows significant differences in the content of macrophages and neutrophils, indicating a longer and more severe inflammation in the group receiving BLM sulfate, which suggests that with longer observation (over 21 days), BLM sulfate may cause more severe fibrosis.

## Figures and Tables

**Table 1:** Experimental design using the BLM-induced PF model.

Group Number	Group Description	BLM Dose	Drug	Number of Animals
1	SHAM	0	Vehicle	5
2	BLM	3 mg/kg	Vehicle	5
3	BLM	3 mg/kg	Test article	5
4	BLM	3 mg/kg	Comparison article	5

**Table 2:** A selection of publications on modeling PF in rats and mice, in which there is an indication of the BLM manufacturer and/or the ability to determine which particular form of BLM was used (source PubMed search engine).

#№	BLM Salt (Hydrochloride or Sulfate)	BLM Form (Reagent or Pharmaceutical)	Manufacturer	Type of Animal (Mice or Rat), Dose	Reference
1	Hydrochloride	Pharmaceutical drug	ZHEJIANG HISUN PHARMACEUTICAL Co., Ltd. Bleomycin chloride	Rats, 5 mg/kg	[10]
2	Sulfate	Reagent	630-107-M010, Enzo Life Sciences, USA	Rats, 2.5 mg/kg	[16]
3	Hydrochloride	Pharmaceutical drug	Nippon Kayaku Co., Ltd. (Tokyo, Japan).	Rats, 5 mg/kg	[17]
4	Hydrochloride	Pharmaceutical drug	Nippon Kayaku Co., Ltd. (Tokyo, Japan).	Rats, 5 mg/kg	[18]
5	Hydrochloride	Pharmaceutical drug	Zhejiang Hai Zheng Pharmaceuticals	Rats, 5 mg/kg	[19]
6	Hydrochloride	Pharmaceutical drug	Nippon Kayaku Co., Ltd. (Tokyo, Japan).	Rats, 3 mg/kg	[9]
7	Sulfate	Reagent	Merck, Darmstadt, Germany	Rats, 5 mg/kg	[20]
8	Hydrochloride	Pharmaceutical drug	Cell Pharm GmbH (Bad Vilbel, Germany)	Rats, 5 mg/kg	[21]
9	Sulfate	Reagent	Sigma, USA	Rats, 2 mg/kg	[22]
10	Hydrochloride	Pharmaceutical drug	Bleocip 15IU, Cipla Pharmaceutical Company, Mumbai, India	Rats, 5 mg/kg	[7]
11	Hydrochloride	Pharmaceutical drug	Zhejiang Hai Zheng Pharmaceuticals	Mice, 3 mg/kg	[6]
12	Sulfate	Reagent	Sigma chemical company, USA	Mice	[23]
13	Sulfate	Pharmaceutical drug	SICOR Pharmaceuticals, Inc., Irvine, CA or TEVA	Mice, 0.8 mg/kg	[24]
14	Hydrochloride	Pharmaceutical drug	Nippon Kayaku, Japan, H20090885	Mice, 2 mg/kg	[25]
15	Hydrochloride	Pharmaceutical drug	Hanhui Pharmaceutical Co., Ltd., Shanghai, China	Mice, 3 mg/kg, 5 mg/kg	[8]
16	Hydrochloride	Pharmaceutical drug	Baxter Healthcare GmbH, 1020 Wien	Mice, 0.3 mg/kg	[26]
17	Sulfate	Pharmaceutical drug	Hospira, Melbourne, VIC, Australia	Mice, 1 mg/kg	[27]
18	Sulfate	Reagent	B1141000, Sigma-Aldrich Inc.	Mice, 4 mg/kg	[28]
19	Sulfate	Reagent	Sigma-Aldrich Inc.	Mice 2 mg/kg	[29]
20	Hydrochloride	Pharmaceutical drug	Nippon Kayaku, Japan, H20090885	Mice, 3 mg/kg	[30]
21	Hydrochloride	Pharmaceutical drug	Nippon Kayaku, Japan, H20090885	Mice, 2 mg/kg	[31]
22	Sulfate	Pharmaceutical drug	Hisun-Pfizer Pharmaceuticals, Shanghai, China	Mice, 5мг	[32]
23	Sulfate	Not indicated	Not indicated	Mice, 3 mg/kg	[33]
24	Sulfate	Not indicated	Not indicated	Mice, 3 mg/kg	[34]
25	Hydrochloride	Pharmaceutical drug	Nippon Kayaku, Japan, H20090885	Mice, 3 mg/kg	[35]
26	Sulfate	Reagent	Sigma-Aldrich Inc.	Mice, 3 mg/kg	[36]
27	Hydrochloride	Reagent	MedChemExpress, HY-17565A	Mice, 5 mg/kg	[37]
28	Hydrochloride	Pharmaceutical drug	Baxter Healthcare GmbH, 1020 Wien	Mice, 1.25 mg/kg	[38]
29	Hydrochloride	Pharmaceutical drug	Nippon Kayaku, Japan, H20090885	Mice, 4 mg/kg	[39]
30	Sulfate	Reagent	Selleck, Shanghai, China	Mice, 5 mg/kg	[40]
31	Sulfate	Reagent	MedChemExpress, New Jersey, USA	Mice, 1.5 mg/kg	[41]
32	Hydrochloride	Pharmaceutical drug	Nippon Kayaku, Japan	Mice, 1.7 mg/kg	[42]
33	Hydrochloride	Pharmaceutical drug	Baxter Healthcare GmbH, 1020 Wien	Mice, 1 mg/kg	[43]
34	Sulfate	Reagent	Merck, Darmstadt, Germany	Mice, 1.5 mg/kg	[44]
35	Sulfate	Reagent	GlpBio Technology Inc. (Montclair, CA, USA)	Mice, 3 mg/kg	[45]
36	Hydrochloride	Pharmaceutical drug	Hanhui Pharmaceutical Co., Ltd. (Shanghai, China)	Rats, 5 mg/kg	[46]
37	Hydrochloride	Pharmaceutical drug	Hanhui Pharmaceuticals Co., Ltd. (Hangzhou, China)	Rats, 10 mg/kg	[47]
38	Hydrochloride	Pharmaceutical drug	Nippon Kayaku, Japan	Rats, 8 mg/kg	[48]
39	Sulfate	Pharmaceutical drug	Zydus Hospira Oncology Private Ltd., Gujarat, India	Rats, 1.5 mg/kg, 2 mg/kg, 2.5 mg/kg	[49]
40	Hydrochloride	Pharmaceutical drug	Nippon Kayaku Co., Ltd., Japan; Batch No. 440392; Import Drug Registration Certification No. H20090885	Rats, 5 mg/kg	[50]
41	Hydrochloride	Pharmaceutical drug	Hanhui Pharmaceuticals Co., Ltd., batch number 20067411	Rats, 5 mg/kg	[51]
42	Hydrochloride	Pharmaceutical drug	Zhejiang Hisun Pharmaceutical Co., Ltd. (Taizhou, Zhejiang, China).	Rats, 5 mg/kg	[52]
43	Hydrochloride	Pharmaceutical drug	Nippon Kayaku Co., Ltd., Japan	Rats, 5 mg/kg	[53]
44	Sulfate	Reagent	MCE, Dallas, TX, USA	Rats, 5 mg/kg	[54]
45	Sulfate	Not indicated	Not indicated	Rats, 5 mg/kg	[55]
46	Sulfate	Pharmaceutical drug	Cipla Pharmaceuticals, Mumbai, India)	Rats, 5 mg/kg	[56]
47	Sulfate	Reagent	Apollo Scientific Stockport, Cheshire, UK	Rats, 1 mg/kg	[57]
48	Sulfate	Pharmaceutical drug	Sanofi	Rats, 7.5 mg/kg	[58]
49	Hydrochloride	Pharmaceutical drug	Hisun Pharmaceutical Co., Ltd., Zhejiang, China, State Drug Approval Document Number: H20055883	Rats, 5 mg/kg	[59]
50	Sulfate	Reagent	Selleck, S1214	Rats, 5 mg/kg	[60]

**Table 3:** Cytological composition of BALF.

Parameter	Control N = 5	BLM Sulfate N = 5	BLM Chloride N = 5
Total cell concentration ×10^5^/mL	2.57 ± 1.17	14.5 ± 5.29 **	4.65 ± 2.28 ##
Alveolar macrophages, %	98.1 ± 1.1	90.6 ± 2.6 **	92.6 ± 1.4 **
Alveolar macrophages, ×10^5^/mL	2.53 ± 1.17	13.25 ± 5.15 **	4.31 ± 2.11 ##
Band neutrophils, %	0.0 ± 0.0	0.01 ± 0.2	0.0 ± 0.0
Band neutrophils, ×10^5^/mL	0.0 ± 0.0	0.018 ± 0.028	0.0 ± 0.0
Segmented neutrophils, %	0.2 ± 0.2	4.1 ± 1.8 **	2.7 ± 0.9 **##
Segmented neutrophils, ×10^5^/mL	0.005 ± 0.006	0.532 ± 0.151 **	0.117 ± 0.056 **
Eosinophils, %	0.0 ± 0.0	0.3 ± 0.3	0.1 ± 0.2
Eosinophils, ×10^5^/mL	0.00 ± 0.00	0.04 ± 0.05	0.01 ± 0.01
Lymphocytes,%	1.7 ± 1.2	4.9 ± 1.6 *	4.6 ± 1.2 *
Lymphocytes, ×10^5^/mL	0.037 ± 0.025	0.663 ± 0.175 **	0.220 ± 0.138 *##

* *p* ≤ 0.05, ** *p* ≤ 0.01 relative to the control group, ## *p* ≤ 0.01 relative to the BLM sulfate group ** *p* ≤ 0.01 relative to the control group in accordance with Mann–Whitney U-test in pairwise comparison. Data are presented as means ± standard deviation.

## Data Availability

All individual data for all animals can be obtained upon request from the corresponding author.
